# Supplementary material for: Predicting Progression of Autosomal Dominant Polycystic Kidney Disease by Changes in the Telomeric Epigenome
Source: Cells. 2022 Oct 20;11(20):3300. doi: 10.3390/cells11203300 (PMC9600909; doi:10.3390/cells11203300)
Supplement: Supplementary file 1 [file cells-11-03300-s001.zip › cells-1907124-supplementary.pdf]

Supp. Table S1. List of genes localized on human chromosome 16p		
**Symbol(↓)**	**Location(↓)**	**Description**
DDX11L10	16p13.3	DEAD/H-box helicase 11 like 10 (pseudogene)
WASIR2	16p13.3	WASH and IL9R antisense RNA 2
POLR3K	16p13.3	RNA polymerase III subunit K
SNRNP25	16p13.3	small nuclear ribonucleoprotein U11/U12 subunit 25
RHBDF1	16p13.3	rhomboid 5 homolog 1
MPG	16p13.3	N-methylpurine DNA glycosylase
NPRL3	16p13.3	NPR3 like, GATOR1 complex subunit
HBZ	16p13.3	hemoglobin subunit zeta
HBM	16p13.3	hemoglobin subunit mu
HBA2	16p13.3	hemoglobin subunit alpha 2
HBA1	16p13.3	hemoglobin subunit alpha 1
HBQ1	16p13.3	hemoglobin subunit theta 1
LUC7L	16p13.3	LUC7 like
FAM234A	16p13.3	family with sequence similarity 234 member A
FAM234A	16p13.3	family with sequence similarity 234 member A
RGS11	16p13.3	regulator of G protein signaling 11
ARHGDIG	16p13.3	Rho GDP dissociation inhibitor gamma
PDIA2	16p13.3	protein disulfide isomerase family A member 2
AXIN1	16p13.3	axin 1
MRPL28	16p13.3	mitochondrial ribosomal protein L28
PGAP6	16p13.3	post-glycosylphosphatidylinositol attachment to proteins 6
NME4	16p13.3	NME/NM23 nucleoside diphosphate kinase 4
DECR2	16p13.3	2,4-dienoyl-CoA reductase 2
RAB11FIP3	16p13.3	RAB11 family interacting protein 3
LINC00235	16p13.3	long intergenic non-protein coding RNA 235
CAPN15	16p13.3	calpain 15
PRR35	16p13.3	proline rich 35
NHLRC4	16p13.3	NHL repeat containing 4
PIGQ	16p13.3	phosphatidylinositol glycan anchor biosynthesis class Q
RAB40C	16p13.3	RAB40C, member RAS oncogene family
WFIKKN1	16p13.3	WAP, follistatin/kazal, immunoglobulin, kunitz and netrin domain containing 1
METTL26	16p13.3	methyltransferase like 26
METTL26	16p13.3	methyltransferase like 26
MCRIP2	16p13.3	MAPK regulated corepressor interacting protein 2
MCRIP2	16p13.3	MAPK regulated corepressor interacting protein 2
WDR90	16p13.3	WD repeat domain 90
RHOT2	16p13.3	ras homolog family member T2
RHBDL1	16p13.3	rhomboid like 1
STUB1	16p13.3	STIP1 homology and U-box containing protein 1
JMJD8	16p13.3	jumonji domain containing 8
WDR24	16p13.3	WD repeat domain 24
FBXL16	16p13.3	F-box and leucine rich repeat protein 16

METRN	16p13.3	meteorin, glial cell differentiation regulator
ANTKMT	16p13.3	adenine nucleotide translocase lysine methyltransferase
CCDC78	16p13.3	coiled-coil domain containing 78
HAGHL	16p13.3	hydroxyacylglutathione hydrolase like
CIAO3	16p13.3	cytosolic iron-sulfur assembly component 3
CIAO3	16p13.3	cytosolic iron-sulfur assembly component 3
MSLN	16p13.3	mesothelin
RPUSD1	16p13.3	RNA pseudouridine synthase domain containing 1
CHTF18	16p13.3	chromosome transmission fidelity factor 18
GNG13	16p13.3	G protein subunit gamma 13
PRR25	16p13.3	proline rich 25
LMF1	16p13.3	lipase maturation factor 1
LMF1-AS1	16p13.3	LMF1 antisense RNA 1
CEROX1	16p13.3	cytoplasmic endogenous regulator of oxidative phosphorylation 1
SOX8	16p13.3	SRY-box transcription factor 8
SSTR5	16p13.3	somatostatin receptor 5
C1QTNF8	16p13.3	C1q and TNF related 8
CACNA1H	16p13.3	calcium voltage-gated channel subunit alpha1 H
TPSG1	16p13.3	tryptase gamma 1
TPSB2	16p13.3	tryptase beta 2
TPSAB1	16p13.3	tryptase alpha/beta 1
TPSD1	16p13.3	tryptase delta 1
UBE2I	16p13.3	ubiquitin conjugating enzyme E2 I
BAIAP3	16p13.3	BAI1 associated protein 3
TSR3	16p13.3	TSR3 ribosome maturation factor
GNPTG	16p13.3	N-acetylglucosamine-1-phosphate transferase subunit gamma
UNKL	16p13.3	unk like zinc finger
C16orf91	16p13.3	chromosome 16 open reading frame 91
PERCC1	16p13.3	proline and glutamate rich with coiled coil 1
CCDC154	16p13.3	coiled-coil domain containing 154
CLCN7	16p13.3	chloride voltage-gated channel 7
PTX4	16p13.3	pentraxin 4
TELO2	16p13.3	telomere maintenance 2
IFT140	16p13.3	intraflagellar transport 140
TMEM204	16p13.3	transmembrane protein 204
CRAMP1	16p13.3	cramped chromatin regulator homolog 1
CRAMP1	16p13.3	cramped chromatin regulator homolog 1
JPT2	16p13.3	Jupiter microtubule associated homolog 2
MAPK8IP3	16p13.3	mitogen-activated protein kinase 8 interacting protein 3
NME3	16p13.3	NME/NM23 nucleoside diphosphate kinase 3
MRPS34	16p13.3	mitochondrial ribosomal protein S34
EME2	16p13.3	essential meiotic structure-specific endonuclease subunit 2

SPSB3	16p13.3	splA/ryanodine receptor domain and SOCS box containing 3
NUBP2	16p13.3	NUBP iron-sulfur cluster assembly factor 2, cytosolic
IGFALS	16p13.3	insulin like growth factor binding protein acid labile subunit
HAGH	16p13.3	Hydroxy acyl glutathione hydrolase
FAHD1	16p13.3	fumarylacetoacetate hydrolase domain containing 1
MEIOB	16p13.3	meiosis specific with OB-fold
LINC00254	16p13.3	long intergenic non-protein coding RNA 254
LINC02124	16p13.3	long intergenic non-protein coding RNA 2124
HS3ST6	16p13.3	heparan sulfate-glucosamine 3-sulfotransferase 6
MSRB1	16p13.3	methionine sulfoxide reductase B1
RPL3L	16p13.3	ribosomal protein L3 like
NDUFB10	16p13.3	NADH:ubiquinone oxidoreductase subunit B10
RPS2	16p13.3	ribosomal protein S2
SNORA10	16p13.3	small nucleolar RNA, H/ACA box 10
SNORA64	16p13.3	small nucleolar RNA, H/ACA box 64
SNHG9	16p13.3	small nucleolar RNA host gene 9
SNORA78	16p13.3	small nucleolar RNA, H/ACA box 78
RNF151	16p13.3	ring finger protein 151
TBL3	16p13.3	transducin beta like 3
NOXO1	16p13.3	NADPH oxidase organizer 1
GFER	16p13.3	growth factor, augmenter of liver regeneration
SYNGR3	16p13.3	synaptogyrin 3
ZNF598	16p13.3	zinc finger protein 598, E3 ubiquitin ligase
NPW	16p13.3	neuropeptide W
SLC9A3R2	16p13.3	SLC9A3 regulator 2
NTHL1	16p13.3	nth like DNA glycosylase 1
TSC2	16p13.3	TSC complex subunit 2
**PKD1**	16p13.3	polycystin 1, transient receptor potential channel interacting
RAB26	16p13.3	RAB26, member RAS oncogene family
SNHG19	16p13.3	small nucleolar RNA host gene 19
SNORD60	16p13.3	small nucleolar RNA, C/D box 60
TRAF7	16p13.3	TNF receptor associated factor 7
CASKIN1	16p13.3	CASK interacting protein 1
MLST8	16p13.3	MTOR associated protein, LST8 homolog
BRICD5	16p13.3	BRICHOS domain containing 5
PGP	16p13.3	phosphoglycolate phosphatase
E4F1	16p13.3	E4F transcription factor 1
DNASE1L2	16p13.3	deoxyribonuclease 1 like 2
ECI1	16p13.3	enoyl-CoA delta isomerase 1
RNPS1	16p13.3	RNA binding protein with serine rich domain 1
ABCA3	16p13.3	ATP binding cassette subfamily A member 3
ABCA17P	16p13.3	ATP binding cassette subfamily A member 17, pseudogene
CCNF	16p13.3	cyclin F

TEDC2	16p13.3	tubulin epsilon and delta complex 2
TEDC2	16p13.3	tubulin epsilon and delta complex 2
NTN3	16p13.3	netrin 3
TBC1D24	16p13.3	TBC1 domain family member 24
ATP6V0C	16p13.3	ATPase H+ transporting V0 subunit c
AMDHD2	16p13.3	amidohydrolase domain containing 2
CEMP1	16p13.3	cementum protein 1
PDPK1	16p13.3	3-phosphoinositide dependent protein kinase 1
LOC652276	16p13.3	potassium channel tetramerization domain containing 5 pseudogene
KCTD5	16p13.3	potassium channel tetramerization domain containing 5
PRSS27	16p13.3	serine protease 27
SRRM2-AS1	16p13.3	SRRM2 antisense RNA 1
SRRM2	16p13.3	serine/arginine repetitive matrix 2
ELOB	16p13.3	elongin B
PRSS33	16p13.3	serine protease 33
SNORA3C	16p13.3	small nucleolar RNA, H/ACA box 3C
PRSS41	16p13.3	serine protease 41
PRSS21	16p13.3	serine protease 21
ZG16B	16p13.3	zymogen granule protein 16B
PRSS22	16p13.3	serine protease 22
FLYWCH2	16p13.3	FLYWCH family member 2
FLYWCH1	16p13.3	FLYWCH-type zinc finger 1
KREMEN2	16p13.3	kringle containing transmembrane protein 2
PAQR4	16p13.3	progestin and adipoQ receptor family member 4
PKMYT1	16p13.3	protein kinase, membrane associated tyrosine/threonine 1
CLDN9	16p13.3	claudin 9
CLDN6	16p13.3	claudin 6
TNFRSF12A	16p13.3	TNF receptor superfamily member 12A
HCFC1R1	16p13.3	host cell factor C1 regulator 1
THOC6	16p13.3	THO complex 6
BICDL2	16p13.3	BICD family like cargo adaptor 2
BICDL2	16p13.3	BICD family like cargo adaptor 2
MMP25	16p13.3	matrix metallopeptidase 25
MMP25-AS1	16p13.3	MMP25 antisense RNA 1
IL32	16p13.3	interleukin 32
ZSCAN10	16p13.3	zinc finger and SCAN domain containing 10
ZNF205	16p13.3	zinc finger protein 205
ZNF213-AS1	16p13.3	ZNF213 antisense RNA 1
ZNF213	16p13.3	zinc finger protein 213
CASP16P	16p13.3	caspase 16, pseudogene
CASP16P	16p13.3	caspase 16, pseudogene
OR1F1	16p13.3	olfactory receptor family 1 subfamily F member 1
OR1F2P	16p13.3	olfactory receptor family 1 subfamily F member 2 pseudogene

ZNF200	16p13.3	zinc finger protein 200
MEFV	16p13.3	MEFV innate immuity regulator, pyrin
LINC00921	16p13.3	long intergenic non-protein coding RNA 921
ZNF263	16p13.3	zinc finger protein 263
TIGD7	16p13.3	tigger transposable element derived 7
ZNF75A	16p13.3	zinc finger protein 75a
OR2C1	16p13.3	olfactory receptor family 2 subfamily C member 1
MTRNR2L4	16p13.3	MT-RNR2 like 4 (pseudogene)
ZSCAN32	16p13.3	zinc finger and SCAN domain containing 32
ZNF174	16p13.3	zinc finger protein 174
ZNF597	16p13.3	zinc finger protein 597
NAA60	16p13.3	N-alpha-acetyltransferase 60, NatF catalytic subunit
C16orf90	16p13.3	chromosome 16 open reading frame 90
CLUAP1	16p13.3	clusterin associated protein 1
NLRC3	16p13.3	NLR family CARD domain containing 3
SLX4	16p13.3	SLX4 structure-specific endonuclease subunit
DNASE1	16p13.3	deoxyribonuclease 1
TRAP1	16p13.3	TNF receptor associated protein 1
CREBBP	16p13.3	CREB binding protein
LINC02861	16p13.3	long intergenic non-protein coding RNA 2861
ADCY9	16p13.3	adenylate cyclase 9
SRL	16p13.3	sarcalumenin
LINC01569	16p13.3	long intergenic non-protein coding RNA 1569
TFAP4	16p13.3	transcription factor AP-4
GLIS2	16p13.3	GLIS family zinc finger 2
GLIS2-AS1	16p13.3	GLIS2 antisense RNA 1
CORO7-PAM16	16p13.3	CORO7-PAM16 readthrough
PAM16	16p13.3	presequence translocase associated motor 16
CORO7	16p13.3	coronin 7
VASN	16p13.3	vasorin
DNAJA3	16p13.3	DnaJ heat shock protein family (Hsp40) member A3
NMRAL1	16p13.3	NmrA like redox sensor 1
HMOX2	16p13.3	heme oxygenase 2
CDIP1	16p13.3	cell death inducing p53 target 1
C16orf96	16p13.3	chromosome 16 open reading frame 96
UBALD1	16p13.3	UBA like domain containing 1
MGRN1	16p13.3	mahogunin ring finger 1
NUDT16L1	16p13.3	nudix hydrolase 16 like 1
ANKS3	16p13.3	ankyrin repeat and sterile alpha motif domain containing 3
ZNF500	16p13.3	zinc finger protein 500
SEPTIN12	16p13.3	septin 12
SMIM22	16p13.3	small integral membrane protein 22
ROGDI	16p13.3	rogdi atypical leucine zipper
GLYR1	16p13.3	glyoxylate reductase 1 homolog
UBN1	16p13.3	ubinuclein 1

PPL	16p13.3	periplakin
SEC14L5	16p13.3	SEC14 like lipid binding 5
NAGPA	16p13.3	N-acetylglucosamine-1-phosphodiester alpha-N-acetylglucosaminidase
NAGPA-AS1	16p13.3	NAGPA antisense RNA 1
C16orf89	16p13.3	chromosome 16 open reading frame 89
ALG1	16p13.3	ALG1 chitobiosyldiphosphodolichol beta-mannosyltransferase
EEF2KMT	16p13.3	eukaryotic elongation factor 2 lysine methyltransferase
LINC01570	16p13.3	long intergenic non-protein coding RNA 1570
RBFOX1	16p13.3	RNA binding fox-1 homolog 1
TMEM114	16p13.2	transmembrane protein 114
METTL22	16p13.2	methyltransferase 22, Kin17 lysine
ABAT	16p13.2	4-aminobutyrate aminotransferase
TMEM186	16p13.2	transmembrane protein 186
PMM2	16p13.2	phosphomannomutase 2
CARHSP1	16p13.2	calcium regulated heat stable protein 1
USP7	16p13.2	ubiquitin specific peptidase 7
C16orf72	16p13.2	chromosome 16 open reading frame 72
LINC02177	16p13.2	long intergenic non-protein coding RNA 2177
LINC01177	16p13.2	long intergenic non-protein coding RNA 1177
LINC01195	16p13.2	long intergenic non-protein coding RNA 1195
GRIN2A	16p13.2	glutamate ionotropic receptor NMDA type subunit 2A
ATF7IP2	16p13.2-p13.13	activating transcription factor 7 interacting protein 2
LINC01290	16p13.13	long intergenic non-protein coding RNA 1290
EMP2	16p13.13	epithelial membrane protein 2
TEKT5	16p13.13	tektin 5
NUBP1	16p13.13	NUBP iron-sulfur cluster assembly factor 1, cytosolic
TVP23A	16p13.13	trans-golgi network vesicle protein 23 homolog A
CIITA	16p13.13	class II major histocompatibility complex transactivator
DEXI	16p13.13	Dexi homolog
CLEC16A	16p13.13	C-type lectin domain containing 16A
SOCS1	16p13.13	suppressor of cytokine signaling 1
TNP2	16p13.13	transition protein 2
PRM3	16p13.13	protamine 3
PRM2	16p13.13	protamine 2
PRM1	16p13.13	protamine 1
RMI2	16p13.13	RecQ mediated genome instability 2
LITAF	16p13.13	lipopolysaccharide induced TNF factor
SNN	16p13.13	stannin
TXNDC11	16p13.13	thioredoxin domain containing 11
ZC3H7A	16p13.13	zinc finger CCCH-type containing 7A
BCAR4	16p13.13	breast cancer anti-estrogen resistance 4
RSL1D1	16p13.13	ribosomal L1 domain containing 1
GSPT1	16p13.13	G1 to S phase transition 1

NPIPB2	16p13.13	nuclear pore complex interacting protein family member B2
TNFRSF17	16p13.13	TNF receptor superfamily member 17
SNX29	16p13.13-p13.12	sorting nexin 29
CPPED1	16p13.12	calcineurin like phosphoesterase domain containing 1
SHISA9	16p13.12	shisa family member 9
ERCC4	16p13.12	ERCC excision repair 4, endonuclease catalytic subunit
LINC02185	16p13.12	long intergenic non-protein coding RNA 2185
LINC02186	16p13.12	long intergenic non-protein coding RNA 2186
MRTFB	16p13.12	myocardin related transcription factor B
MIR193BHG	16p13.12	MIR193b-365a host gene
LINC02130	16p13.12	long intergenic non-protein coding RNA 2130
PARN	16p13.12	poly(A)-specific ribonuclease
BFAR	16p13.12	bifunctional apoptosis regulator
PLA2G10	16p13.12	phospholipase A2 group X
NPIPA2	16p13.11	nuclear pore complex interacting protein family member A2
NPIPA3	16p13.11	nuclear pore complex interacting protein family member A3
ABCC6P2	16p13.11	ATP binding cassette subfamily C member 6 pseudogene 2
NOMO3	16p13.11	NODAL modulator 3
NOMO1	16p13.11	NODAL modulator 1
PKD1P3-NPIPA1	16p13.11	PKD1P3-NPIPA1 readthrough
NPIPA1	16p13.11	nuclear pore complex interacting protein family member A1
PDXDC1	16p13.11	pyridoxal dependent decarboxylase domain containing 1
NTAN1	16p13.11	N-terminal asparagine amidase
RRN3	16p13.11	RRN3 homolog, RNA polymerase I transcription factor
PKD1P6-NPIPP1	16p13.11	PKD1P6-NPIPP1 readthrough
NPIPA5	16p13.11	nuclear pore complex interacting protein family member A5
NPIPA5	16p13.11	nuclear pore complex interacting protein family member A5
MPV17L	16p13.11	MPV17 mitochondrial inner membrane protein like
BMERB1	16p13.11	bMERB domain containing 1
MARF1	16p13.11	meiosis regulator and mRNA stability factor 1
NDE1	16p13.11	nudE neurodevelopment protein 1
MYH11	16p13.11	myosin heavy chain 11
CEP20	16p13.11	centrosomal protein 20
ABCC1	16p13.11	ATP binding cassette subfamily C member 1
ABCC6	16p13.11	ATP binding cassette subfamily C member 6
NOMO2	16p12.3	NODAL modulator 2
PKD1P4-NPIPA8	16p12.3	PKD1P4-NPIPA8 readthrough
PKD1P1	16p13.11	polycystin 1, transient receptor potential channel interacting pseudogene 1

NPIPA8	16p12.3	nuclear pore complex interacting protein family member A8
NPIPA7	16p13.11	nuclear pore complex interacting protein family member A7
XYLT1	16p12.3	xylosyltransferase 1
PKD1P5-LOC105376752	16p12.3	PKD1P5-LOC105376752 readthrough
ABCC6P1	16p12.3	ATP binding cassette subfamily C member 6 pseudogene 1
RPS15A	16p12.3	ribosomal protein S15a
ARL6IP1	16p12.3	ADP ribosylation factor like GTPase 6 interacting protein 1
SMG1	16p12.3	SMG1 nonsense mediated mRNA decay associated PI3K related kinase
TMC7	16p12.3	transmembrane channel like 7
COQ7	16p12.3	coenzyme Q7, hydroxylase
ITPRIPL2	16p12.3	ITPRIP like 2
SYT17	16p12.3	synaptotagmin 17
CLEC19A	16p12.3	C-type lectin domain containing 19A
TMC5	16p12.3	transmembrane channel like 5
GDE1	16p12.3	glycerophosphodiester phosphodiesterase 1
CCP110	16p12.3	centriolar coiled-coil protein 110
VPS35L	16p12.3	VPS35 endosomal protein sorting factor like
KNOP1	16p12.3	lysine rich nucleolar protein 1
IQCK	16p12.3	IQ motif containing K
GPRC5B	16p12.3	G protein-coupled receptor class C group 5 member B
GPR139	16p12.3	G protein-coupled receptor 139
GP2	16p12.3	glycoprotein 2
UMOD	16p12.3	uromodulin
PDILT	16p12.3	protein disulfide isomerase like, testis expressed
ACSM5	16p12.3	acyl-CoA synthetase medium chain family member 5
ACSM2A	16p12.3	acyl-CoA synthetase medium chain family member 2A
ACSM2B	16p12.3	acyl-CoA synthetase medium chain family member 2B
ACSM1	16p12.3	acyl-CoA synthetase medium chain family member 1
THUMPD1	16p12.3	THUMP domain containing 1
ACSM3	16p12.3	acyl-CoA synthetase medium chain family member 3
ERI2	16p12.3	ERI1 exoribonuclease family member 2
REXO5	16p12.3	RNA exonuclease 5
DCUN1D3	16p12.3	defective in cullin neddylation 1 domain containing 3
LYRM1	16p12.3	LYR motif containing 1
DNAH3	16p12.3	dynein axonemal heavy chain 3
ZP2	16p12.3-p12.2	zona pellucida glycoprotein 2
ANKS4B	16p12.2	ankyrin repeat and sterile alpha motif domain containing 4B
CRYM	16p12.2	crystallin mu
NPIPB3	16p12.2	nuclear pore complex interacting protein family member B3
SMG1P3	16p12.2	SMG1 pseudogene 3

RRN3P3	16p12.2	RRN3 pseudogene 3
METTL9	16p12.2	methyltransferase like 9
IGSF6	16p12.2	immunoglobulin superfamily member 6
OTOA	16p12.2|16p12.2	otoancorin
OTOAP1	16p12.2	OTOA pseudogene 1
RRN3P1	16p12.2	RRN3 pseudogene 1
NPIPB4	16p12.2	nuclear pore complex interacting protein family member B4
NPIPB5	16p12.2	nuclear pore complex interacting protein family member B5
UQCRC2	16p12.2	ubiquinol-cytochrome c reductase core protein 2
PDZD9	16p12.2	PDZ domain containing 9
MOSMO	16p12.2	modulator of smoothened
MOSMO	16p12.2	modulator of smoothened
VWA3A	16p12.2	von Willebrand factor A domain containing 3A
SDR42E2	16p12.2	short chain dehydrogenase/reductase family 42E, member 2
EEF2K	16p12.2	eukaryotic elongation factor 2 kinase
POLR3E	16p12.2	RNA polymerase III subunit E
CDR2	16p12.2	cerebellar degeneration related protein 2
MFSD13B	16p12.2	major facilitator superfamily domain containing 13B (pseudogene)
HS3ST2	16p12.2	heparan sulfate-glucosamine 3-sulfotransferase 2
USP31	16p12.2	ubiquitin specific peptidase 31
SCNN1G	16p12.2	sodium channel epithelial 1 subunit gamma
SCNN1B	16p12.2	sodium channel epithelial 1 subunit beta
COG7	16p12.2	component of oligomeric golgi complex 7
GGA2	16p12.2	golgi associated, gamma adaptin ear containing, ARF binding protein 2
EARS2	16p12.2	glutamyl-tRNA synthetase 2, mitochondrial
UBFD1	16p12.2	ubiquitin family domain containing 1
NDUFAB1	16p12.2	NADH:ubiquinone oxidoreductase subunit AB1
PALB2	16p12.2	partner and localizer of BRCA2
DCTN5	16p12.2	dynactin subunit 5
PLK1	16p12.2	polo like kinase 1
ERN2	16p12.2	endoplasmic reticulum to nucleus signaling 2
CHP2	16p12.2	calcineurin like EF-hand protein 2
PRKCB	16p12.2-p12.1	protein kinase C beta
LINC02194	16p12.1	long intergenic non-protein coding RNA 2194
CACNG3	16p12.1	calcium voltage-gated channel auxiliary subunit gamma 3
RBBP6	16p12.1	RB binding protein 6, ubiquitin ligase
LINC01567	16p12.1	long intergenic non-protein coding RNA 1567
TNRC6A	16p12.1	trinucleotide repeat containing adaptor 6A
SLC5A11	16p12.1	solute carrier family 5 member 11
ARHGAP17	16p12.1	Rho GTPase activating protein 17

LOC554206	16p12.1	leucine carboxyl methyltransferase 1 pseudogene
LINC02175	16p12.1	long intergenic non-protein coding RNA 2175
LCMT1-AS1	16p12.1	LCMT1 antisense RNA 1
LCMT1	16p12.1	leucine carboxyl methyltransferase 1
LCMT1-AS2	16p12.1	LCMT1 antisense RNA 2
AQP8	16p12.1	aquaporin 8
ZKSCAN2	16p12.1	zinc finger with KRAB and SCAN domains 2
HS3ST4	16p12.1	heparan sulfate-glucosamine 3-sulfotransferase 4
C16orf82	16p12.1	chromosome 16 open reading frame 82
KDM8	16p12.1	lysine demethylase 8
NSMCE1	16p12.1	NSE1 homolog, SMC5-SMC6 complex component
NSMCE1-DT	16p12.1	NSMCE1 divergent transcript
IL4R	16p12.1	interleukin 4 receptor
IL21R	16p12.1	interleukin 21 receptor
IL21R-AS1	16p12.1	IL21R antisense RNA 1
GTF3C1	16p12.1	general transcription factor IIIC subunit 1
GSG1L	16p12.1	GSG1 like
XPO6	16p12.1	exportin 6
SBK1	16p12.1	SH3 domain binding kinase 1
NPIPB6	16p12.1	nuclear pore complex interacting protein family member B6
EIF3C	16p11.2	eukaryotic translation initiation factor 3 subunit C
EIF3CL	16p12.1	eukaryotic translation initiation factor 3 subunit C like
CLN3	16p12.1	CLN3 lysosomal/endosomal transmembrane protein, battenin
APOBR	16p12.1	apolipoprotein B receptor
IL27	16p12.1-p11.2	interleukin 27
NUPR1	16p11.2	nuclear protein 1, transcriptional regulator
SGF29	16p11.2	SAGA complex associated factor 29
SGF29	16p11.2	SAGA complex associated factor 29
SULT1A2	16p11.2	sulfotransferase family 1A member 2
SULT1A1	16p11.2	sulfotransferase family 1A member 1
NPIPB8	16p11.2	nuclear pore complex interacting protein family member B8
NPIPB9	16p11.2	nuclear pore complex interacting protein family member B9
ATXN2L	16p11.2	ataxin 2 like
TUFM	16p11.2	Tu translation elongation factor, mitochondrial
SH2B1	16p11.2	SH2B adaptor protein 1
ATP2A1	16p11.2	ATPase sarcoplasmic/endoplasmic reticulum Ca2+ transporting 1
ATP2A1-AS1	16p11.2	ATP2A1 antisense RNA 1
RABEP2	16p11.2	rabaptin, RAB GTPase binding effector protein 2
CD19	16p11.2	CD19 molecule
NFATC2IP	16p11.2	nuclear factor of activated T cells 2 interacting protein
SPNS1	16p11.2	sphingolipid transporter 1 (putative)

LAT	16p11.2	linker for activation of T cells
RRN3P2	16p11.2	RRN3 pseudogene 2
SNX29P2	16p11.2	sorting nexin 29 pseudogene 2
NPIPB11	16p11.2	nuclear pore complex interacting protein family member B11
SMG1P6	16p11.2	SMG1 pseudogene 6
BOLA2-SMG1P6	16p11.2	BOLA2-SMG1P6 readthrough
LOC606724	16p11.2	coronin 1A pseudogene
BOLA2	16p11.2	bolA family member 2
BOLA2B	16p11.2	bolA family member 2B
SLX1A	16p11.2	SLX1 homolog A, structure-specific endonuclease subunit
SLX1B	16p11.2	SLX1 homolog B, structure-specific endonuclease subunit
SLX1A-SULT1A3	16p11.2	SLX1A-SULT1A3 readthrough (NMD candidate)
SLX1B-SULT1A4	16p11.2	SLX1B-SULT1A4 readthrough (NMD candidate)
SULT1A3	16p11.2	sulfotransferase family 1A member 3
SULT1A4	16p11.2	sulfotransferase family 1A member 4
LOC388242	16p11.2	SAGA complex associated factor 29 pseudogene
LOC613038	16p11.2	SAGA complex associated factor 29 pseudogene
NPIPB12	16p11.2	nuclear pore complex interacting protein family member B12
SMG1P2	16p11.2	SMG1 pseudogene 2
SPN	16p11.2	sialophorin
QPRT	16p11.2	quinolinate phosphoribosyltransferase
C16orf54	16p11.2	chromosome 16 open reading frame 54
ZG16	16p11.2	zymogen granule protein 16
KIF22	16p11.2	kinesin family member 22
MAZ	16p11.2	MYC associated zinc finger protein
PRRT2	16p11.2	proline rich transmembrane protein 2
PAGR1	16p11.2	PAXIP1 associated glutamate rich protein 1
MVP	16p11.2	major vault protein
CDIPT	16p11.2	CDP-diacylglycerol--inositol 3-phosphatidyltransferase
CDIPTOSP	16p11.2	CDIP transferase opposite strand, pseudogene
CDIPTOSP	16p11.2	CDIP transferase opposite strand, pseudogene
SEZ6L2	16p11.2	seizure related 6 homolog like 2
ASPHD1	16p11.2	aspartate beta-hydroxylase domain containing 1
KCTD13	16p11.2	potassium channel tetramerization domain containing 13
TMEM219	16p11.2	transmembrane protein 219
TAOK2	16p11.2	TAO kinase 2
HIRIP3	16p11.2	HIRA interacting protein 3
INO80E	16p11.2	INO80 complex subunit E
DOC2A	16p11.2	double C2 domain alpha
C16orf92	16p11.2	chromosome 16 open reading frame 92
TLCD3B	16p11.2	TLC domain containing 3B
ALDOA	16p11.2	aldolase, fructose-bisphosphate A
PPP4C	16p11.2	protein phosphatase 4 catalytic subunit

TBX6	16p11.2	T-box transcription factor 6
YPEL3	16p11.2	yippee like 3
GDPD3	16p11.2	glycerophosphodiester phosphodiesterase domain containing 3
MAPK3	16p11.2	mitogen-activated protein kinase 3
CORO1A	16p11.2	coronin 1A
NPIPB13	16p11.2	nuclear pore complex interacting protein family, member B13
CD2BP2	16p11.2	CD2 cytoplasmic tail binding protein 2
TBC1D10B	16p11.2	TBC1 domain family member 10B
MYLPF	16p11.2	myosin light chain, phosphorylatable, fast skeletal muscle
SEPTIN1	16p11.2	septin 1
ZNF48	16p11.2	zinc finger protein 48
ZNF771	16p11.2	zinc finger protein 771
SNORA80C	16p11.2	small nucleolar RNA, H/ACA box 80C
DCTPP1	16p11.2	dCTP pyrophosphatase 1
SEPHS2	16p11.2	selenophosphate synthetase 2
ITGAL	16p11.2	integrin subunit alpha L
ZNF768	16p11.2	zinc finger protein 768
ZNF747	16p11.2	zinc finger protein 747
ZNF764	16p11.2	zinc finger protein 764
ZNF688	16p11.2	zinc finger protein 688
ZNF785	16p11.2	zinc finger protein 785
ZNF689	16p11.2	zinc finger protein 689
PRR14	16p11.2	proline rich 14
FBRS	16p11.2	fibrosin
SRCAP	16p11.2	Snf2 related CREBBP activator protein
SNORA30	16p11.2	small nucleolar RNA, H/ACA box 30
TMEM265	16p11.2	transmembrane protein 265
PHKG2	16p11.2	phosphorylase kinase catalytic subunit gamma 2
RNF40	16p11.2	ring finger protein 40
ZNF629	16p11.2	zinc finger protein 629
MIR762HG	16p11.2	MIR762 host gene
BCL7C	16p11.2	BAF chromatin remodeling complex subunit BCL7C
CTF1	16p11.2	cardiotrophin 1
FBXL19	16p11.2	F-box and leucine rich repeat protein 19
ORAI3	16p11.2	ORAI calcium release-activated calcium modulator 3
SETD1A	16p11.2	SET domain containing 1A, histone lysine methyltransferase
HSD3B7	16p11.2	hydroxy-delta-5-steroid dehydrogenase, 3 beta- and steroid delta-isomerase 7
STX1B	16p11.2	syntaxin 1B
STX4	16p11.2	syntaxin 4
ZNF668	16p11.2	zinc finger protein 668
ZNF646	16p11.2	zinc finger protein 646
PRSS53	16p11.2	serine protease 53

VKORC1	16p11.2	vitamin K epoxide reductase complex subunit 1
BCKDK	16p11.2	branched chain keto acid dehydrogenase kinase
KAT8	16p11.2	lysine acetyltransferase 8
PRSS8	16p11.2	serine protease 8
PRSS36	16p11.2	serine protease 36
FUS	16p11.2	FUS RNA binding protein
PYCARD	16p11.2	PYD and CARD domain containing
PYCARD-AS1	16p11.2	PYCARD antisense RNA 1
TRIM72	16p11.2	tripartite motif containing 72
PYDC1	16p11.2	pyrin domain containing 1
ITGAM	16p11.2	integrin subunit alpha M
ITGAX	16p11.2	integrin subunit alpha X
ITGAD	16p11.2	integrin subunit alpha D
COX6A2	16p11.2	cytochrome c oxidase subunit 6A2
ZNF843	16p11.2	zinc finger protein 843
ARMC5	16p11.2	armadillo repeat containing 5
TGFB1I1	16p11.2	transforming growth factor beta 1 induced transcript 1
SLC5A2	16p11.2	solute carrier family 5 member 2
RUSF1	16p11.2	RUS family member 1
AHSP	16p11.2	alpha hemoglobin stabilizing protein
LINC02190	16p11.2	long intergenic non-protein coding RNA 2190
FRG2KP	16p11.2	FSHD region gene 2 family member K, pseudogene
YBX3P1	16p11.2	Y-box binding protein 3 pseudogene 1
VN1R3	16p11.2	vomeronasal 1 receptor 3
ZNF267	16p11.2	zinc finger protein 267
LOC102723753	-	HECT and RLD domain containing E3 ubiquitin protein ligase 2 pseudogene
HERC2P4	16p11.2	HERC2 pseudogene 4
TP53TG3D	16p11.2	TP53 target 3D
LOC390705	16p11.2	protein phosphatase 2 regulatory subunit B'', beta pseudogene
LOC102723655	-	TP53-target gene 3 protein
TP53TG3E	16p11.2	TP53 target 3 family member E
TP53TG3	16p11.2	TP53 target 3
TP53TG3B	16p11.2	TP53 target 3B
TP53TG3F	16p11.2	TP53 target 3 family member F
TP53TG3C	16p11.2	TP53 target 3C
ENPP7P13	16p11.2	ectonucleotide pyrophosphatase/phosphodiesterase 7 pseudogene 13
CCNYL3	16p11.2	cyclin Y like 3 (pseudogene)
UBE2MP1	16p11.2	ubiquitin conjugating enzyme E2 M pseudogene 1
LINC01566	16p11.1	long intergenic non-protein coding RNA 1566
FRG2DP	16p11.1	FSHD region gene 2 family member D, pseudogene
TP53TG3HP	16p11.1	TP53 target 3 family member H, pseudogene
LINC02167	16p11.1	long intergenic non-protein coding RNA 2167
